# MapNext: a software tool for spliced and unspliced alignments and SNP detection of short sequence reads

**DOI:** 10.1186/1471-2164-10-S3-S13

**Published:** 2009-12-03

**Authors:** Hua Bao, Yuanyan Xiong, Hui Guo, Renchao Zhou, Xuemei Lu, Zhen Yang, Yang Zhong, Suhua Shi

**Affiliations:** 1 State Key Laboratory of Biocontrol, School of Life Sciences, Sun Yat-Sen University, Guangzhou, 510275, PR China; 2 School of Life Sciences, Fudan University, Shanghai, 200433, PR China

## Abstract

**Background:**

Next-generation sequencing technologies provide exciting avenues for studies of transcriptomics and population genomics. There is an increasing need to conduct spliced and unspliced alignments of short transcript reads onto a reference genome and estimate minor allele frequency from sequences of population samples.

**Results:**

We have designed and implemented MapNext, a software tool for both spliced and unspliced alignments of short sequence reads onto reference sequences, and automated SNP detection using neighbourhood quality standards. MapNext provides four main analyses: (i) unspliced alignment and clustering of reads, (ii) spliced alignment of transcript reads over intron boundaries, (iii) SNP detection and estimation of minor allele frequency from population sequences, and (iv) storage of result data in a database to make it available for more flexible queries and for further analyses. The software tool has been tested using both simulated and real data.

**Conclusion:**

MapNext is a comprehensive and powerful tool for both spliced and unspliced alignments of short reads and automated SNP detection from population sequences. The simplicity, flexibility and efficiency of MapNext makes it a valuable tool for transcriptomic and population genomic research.

## Background

Next-generation sequencing technologies, based on sequencing by synthesis (SBS), are starting to deliver a large number of DNA sequences at a relatively low cost, thus opening new areas of genomic research. One of the ambitious goals for these technologies is to sequence a complete human genome in several days at a price of $100,000, and eventually $1,000. To this end, sequencing throughput must be increased dramatically. This may be achieved by carrying out many parallel reactions. Despite the fact that the read-length is short (down to 25-50 bp), the overall throughput is enormous, each run producing up to several million reads and billions of base-pairs of sequence data.

While the promise of next-generation sequencing technologies has become a reality, they also present substantial challenges such as in the mapping of short sequence reads to the genome, polymorphism detection, characterization of allele frequencies from population samples and data management. So far, most efforts have been spent in developing methods for unspliced mapping of short sequence reads, and several software tools have been developed. An alignment integrated tool in the Illumina-Solexa data processing package, ELAND, optimizes mapping of very short reads and allows at most only two mismatches between the read and the genomic sequence. MAQ is another program for ungapped alignment with probability models to measure alignment quality [1]. SOAP and SeqMap can conduct both ungapped and gapped alignment for Solexa reads [2,3]. SHRiMP is a package for mapping short reads to highly polymorphic genomes with a statistical method for scoring the alignment [4]. It is required to quickly and accurately align the sequence reads over exon/intron boundaries in transcriptome sequencing, but these programs are not designed for spliced alignments. Most existing programs for spliced alignment such as BLAT and GMAP were designed for mapping Sanger capillary mRNA or EST sequence to genome [5,6] and do not yet have efficient support for short sequence reads. QPalma is specifically designed to align the short sequence reads over intron boundaries [7], but it requires training sets of spliced reads with sequencing quality and known alignments information. Moreover, the existing programs usually detect SNPs in a single individual, and cannot be applied to population samples where high-quality SNPs and allele frequencies require characterization.

Here we provide a freely available software tool, MapNext, for both spliced and unspliced alignments of the short reads and automated SNP detection from population sequences. For spliced alignments, a training process is not needed. Additionly, MapNext is capable of facilitating the conversion of text based sequences and quality output, mapping results and SNP results into a more flexible SQL database.

## Implementation

The main program of MapNext was developed in C++. Some Perl scripts were written to format data, and an SQL script was used to create tables and load the results into the tables in the database. FASTA format for the reference sequences, and either FASTA or FASTQ format for query reads are allowed in the input of MapNext. The current version is designed for Linux/Unix systems. Compiling the program requires a recent version of g++. The implementation of MapNext1.0, full documentation and a downloadable test dataset are freely available [8].

## Results and discussion

### Unspliced alignment

Many of the short sequence mapping programs use k-mer and hash index table algorithm to accelerate alignment. SeqMap and MAQ build k-mer index table for reads, and SOAP builds index table for reference genome. To admit two mismatches, these programs split every read into four fragments and use all six combinations of the two fragments as seed. SHRiMP starts with a rapid k-mer hashing step and executes a vectorized Smith-Waterman step to score and validate the alignment. MapNext also uses a fast k-mer scan to locate the regions of potential homology. But it uses the filtration method described by [9]. For reads of length m, and mapping with up to n mismatches, each read is partitioned n+1 contiguous seeds. So there is at least one seed with no mismatches. MapNext processes the set of query reads to build a hash table indexed by seed. For every seed, its location in the query reads is listed where it occurs.

MapNext scans the reference sequence using a sliding window of size equals to k-mer size at steps of size 1. If the k-mer is a key in the query hash table, the corresponding reads with full length must be compared with adjacent regions of the reference surrounding the k-mer. MapNext counts mismatches between the reads and the reference sequence and stores the corresponding read name, the strand of the read, the number of mismatches and the position of the reference sequence if the number of mismatches does not exceed the maximum number of mismatches assigned by the user. Once the reads have been mapped onto the reference sequences, their locations can be used to precisely map reads to clusters based on the overlaps in the reference. For multiple hits of one read, the program randomly reports one of the hits. The output is given in a tab-separated format containing the reference and query sequence names, the start and end position of the alignment in the reference sequences, as well as the strand and the number of mismatches per read.

### Spliced alignment

Most existing programs were designed for mapping mRNA or EST sequence to genome. QPalma is specifically designed to align the short sequence reads over intron boundaries. Qpalma first needs a dataset of known splice site and a splice site prediction model. It extends Smith-Waterman alignment to take qulity score, splice site and intron length into account. The prediction and training process is time-consuming, and the running steps are complicated.

MapNext first conducts the unspliced alignment of all query reads, then conducts the spliced alignment of reads that cannot be aligned at the first stage. It needs no prediction and traning process. For spliced alignment, MapNext uses two different strategies. One strategy is to generate all potential genomic sequences overlapping with two consecutive exon boundaries (according to annotation of the exons). Since all the introns have been spliced out, the reads left in the first stage can be aligned against these sequences to find the alignment over intron/exon boundaries. It is very efficient to map in this way. The perl script was written to build a file that contains intron-spanning sequences and their position relative to the genome sequences.

Unfortunately, this strategy cannot find un-annotated and novel transcripts such as those from alternative splicing. The other strategy (*de novo*), which is designed to find potential spliced alignments without annotation information, proceeds through the following steps: (1) building a hash table of k-mer on the head and tail of the query reads; (2) fast k-mer scanning to find all genomic locations from which the reads may derive; (3) searching the paired k-mer locations within 10 kbp and with the same strand; (4) extending the alignments in both 5' and 3' directions; and (5) verifying the location of the four boundaries (start and end of two consecutive exons) by the presence of the consensus (forward:GT/AG reverse: CT/AC) splice sites on the genomic locations.

### SNP detection and minor allele frequency estimation

Most existing programs usually detect SNPs in a single individual, and cannot be applied to population samples where high-quality SNPs and allele frequencies require characterization. MapNext provides useful information about the SNP detection from population sequences. Methods of SNP detection can be categorized as two kinds. One is to develop an explicit statistical framework to model variants. The other is based on some heuristic rules. Quality control and significance threshold are the most adopted rules. MapNext uses these rules to detect SNPs from population sequences.

MapNext looks at each position in the cluster to determine if there is an SNP at that position. To make a qualified assessment, it also considers the general quality of the bases around this position [10,11]. Potential nucleotide variants should be greater than the sequence quality value (QV), neighbour quality value (NQV), minimum coverage (MC), and minimum minor allele frequency (MMAF). The default QV value is >25 for the polymorphic base and >20 for the minimal NQV (4 bp). If SNPs were called in areas of low coverage, a higher amount of false positives would occur. Therefore, MC for the SNP to be called should be set. The coverage is counted as the number of valid reads at the current position. For population sequences, the call of minor frequency alleles is very likely affected by sequencing errors. MMAF can be used for determining the minimum frequency of the minor allele of the site to be called an SNP. MapNext calculates MAF from the major and minor allele counts at the SNP site.

To summarize, based on the user's specifications on what will be considered a valid SNP, SNP detection scans through the entire clustering results and report all SNPs that meet the requirements. The output contains the nucleotide bases of major and minor alleles, minor allele frequencies, coverage at the SNP site and the SNP position on the reference sequence.

### Storage of the results

The last role of MapNext is to facilitate the conversion of text-based sequences, quality scores, alignment, and SNP results into a SQL database. The database created with MySQL records the sequences and results in relational tables. An SQL script is written to help users create the database and load data. Users can access the data through a command line client within the Linux server. A web interface is also being implemented to query and visualize the results.

### Accuracy measure

To measureme the accuracy of the alignment algorithm, 1893118 reads(35 bp length), with 0.5% sequencing error rate, from 5796 coding DNA sequences of the chromosome I of *Arabidopsis thaliana *were simulated, in which about 7% of the total reads were spliced (134274 reads). Table 1 shows a comparison of accuracy and running time among the short reads alignment programs, *i.e.*, SHRiMP, SeqMap, SOAP and MAQ for the alignment of the unspliced reads only, and QPalma for the alignment of the spliced reads. MapNext which incorporates both unspliced and spliced alignment of short transcript sequences can map more reads than other programs. Moreover, MapNext can achieve nearly the same sensitivity and accuracy for spliced alignment without a training process. For unspliced alignment, MapNext is faster than SHRiMP and SeqMap, but slower than SOAP and MAQ. For spliced alignment, MapNext is faster than Qpalma.

**Table 1:** Benchmark results of short reads alignment programs.

	Unspliced alignment			Spiced alignment		
	
Program	True positive (%)	False positive (%)	Running time (s)	True positive (%)	False positive (%)	Running time (m)
SHRiMP	94.79	8.97	809	N/A	N/A	N/A
SeqMap	96.50	6.71	447	N/A	N/A	N/A
SOAP	96.41	6.72	101	N/A	N/A	N/A
MAQ	96.53	6.73	138	N/A	N/A	N/A
Qpalma	N/A	N/A	N/A	85.17	4.45	557

MapNext	96.51	6.72	209	83.89	4.31	231

A total of 1893118 reads (35 bp length, 134274 spliced and 1758844 unspliced) from 5796 coding DNA sequences of chromosome I of *Arabidopsis thaliana *for the query dataset were simulated. Accuracy measures were calculated under the same threshold by allowing at most two mismatches. For the unspliced alignment, the true and false positive rates equal the number of short reads with correct and incorrect alignment positions divided by 1758844, respectively. For the spliced alignment, the true and false positive rates equal the number of short reads with correct and incorrect alignment positions divided by 134274.

**Figure 1:**
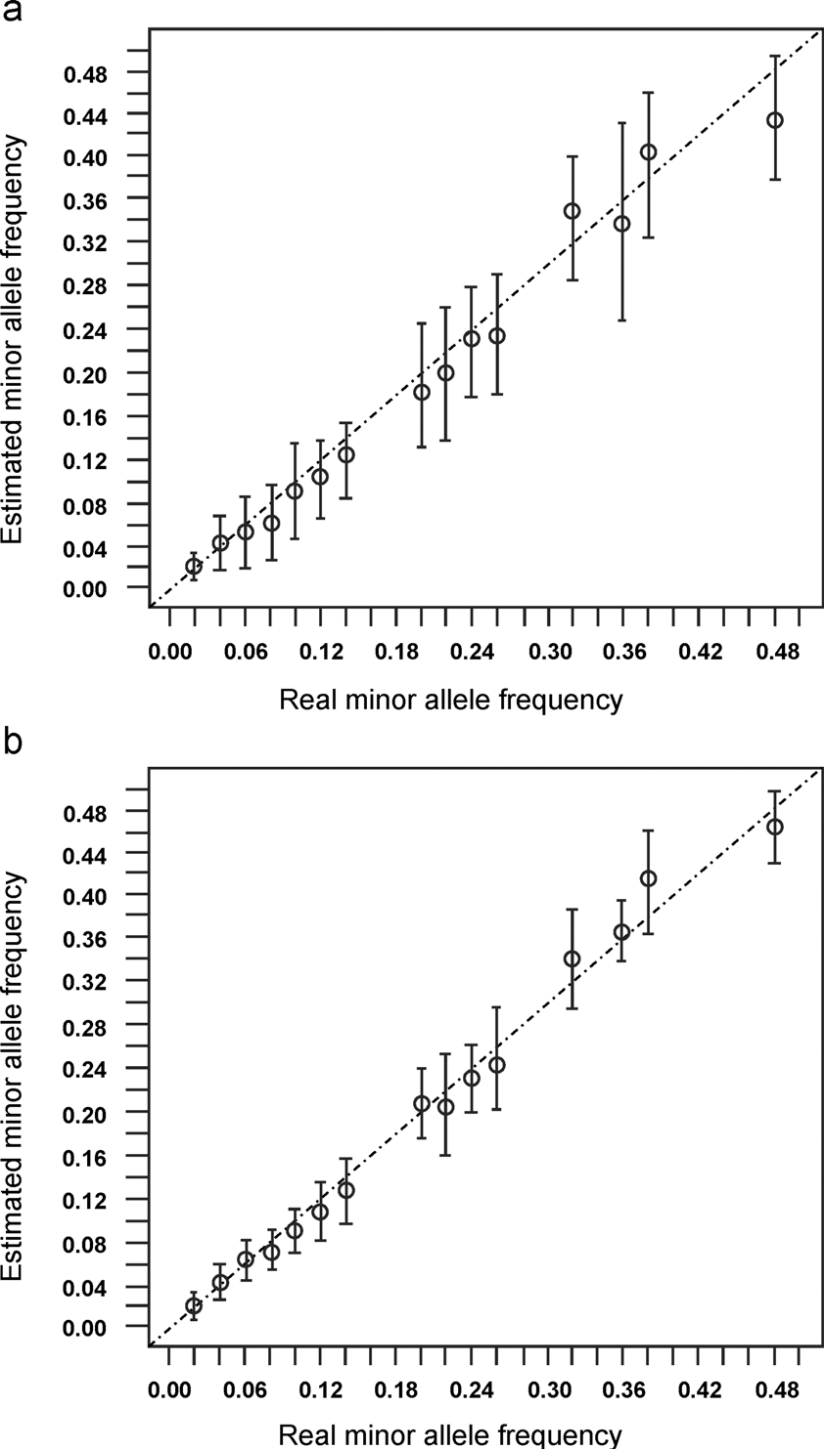
**The accuracy of minor allele frequency estimation produced by MapNext**. The mean (SD) of minor allele frequencies are given. QV, NQV, MMAF and MC are set 25, 20, 0.01 and 50 respectively. (a) The sequencing coverage is 2× per individual. (b) The sequencing coverage is 12× per individual.

To measureme the accuracy of SNP detection in population sequences, a set of short reads data from population sequences known as true SNPs and their frequencies was simulated. Population sequences (sequence length = 100000, sample size = 50 and Theta = 0.005) were generated under a neutral evolution model using ms [12] and Seq-Gen [13] software. The population sequences were then randomly split into different numbers of 35 bp short reads and the empirical quality scores were added for each site. The empirical quality scores were obtained from sequence reads generated by Illumina-Solexa Genome Analyzer. Sequencing errors were added to these sites according to the probability of sequencing errors (Q = -10 * log(e/(1 - e)), where Q is the quality score, and e is the probability of sequencing error). One sequence was randomly chosen from the population sequences as the reference sequence for alignment. Mapping and SNP detection from those short reads was performed with MapNext. The candidate SNPs were compared with real SNPs to evaluate the accuracy of SNP calling. Table 2 shows true positive and false positive rates at various levels of read coverage for SNP detection. At a coverage of 8X, for example, the SNP true positive rate can be as high as 93.20%, while the false positive rate is less than 0.5%. The accuracy of estimation of allele frequencies was also evaluated from the estimated data by comparison to real frequencies data (Figure 1).  The SD of the frequency esetimation is smaller at high coverage.

**Table 2:** Accuracy of SNP detection produced by MapNext.

Coverage	True Positives	False Positives
4×	1961 (90.70%)	690 (29.51%)
6×	1998 (92.41%)	23 (1.06%)
8×	2015 (93.20%)	8 (0.37%)
10×	2043 (94.50%)	0 (0.00%)
12×	2068 (95.65%)	0 (0.00%)

There were 2162 true SNPs in 50 individuals (haploid) in our simulation. Coverage equals sequencing depth per individual. QV, NQV, MMAF and MC were set at 25, 20, 0.01 and 50 (1× per individual), respectively.

A real dataset from *Sonneratia alba*, a mangrove species widely distributed in the western Indo-Pacific region was used to test our program. Seventy-five nuclear genes were sequenced from a sample that was a mixture of 100 individuals of *S. alba*. The query dataset contained 8161413 single-end reads (length 35 bp) generated by Illumina-Solexa Genome Analyzer. Out of these reads, 5695624 were mapped onto the reference sequences. Finally, a total of 30 putative SNPs were found and their allele frequencies were predicted.

## Conclusion

MapNext is a comprehensive and powerful tool for both spliced and unspliced alignment of the short reads onto reference sequences and SNP detection from population sequences. It can facilitate the conversion of text based output into a SQL database, allowing users to query for results easily. MapNext enables researchers to efficiently analyze data in the study of transcriptomics and population genomics.

## Availability and requirements

Project name: MapNext 1.0

Project home page: http://evolution.sysu.edu.cn/english/software/mapnext.htm

Operating system(s): Linux

Programming language: C++, Perl and MySQL

Other requirements: MySQL 5.0

License: Academic software license must be agreed upon during installation

Any restrictions to use by non-academics: Yes

## Competing interests

The authors declare that they have no competing interests.

## Authors' contributions

HB designed the algorithms and database, coded and implemented the entire MapNext 1.0 and drafted the manuscript. YX simulated the data, evaluated the performance of the program, wrote the C++ code for spliced alignment and revised the manuscript. HG wrote the C++ code for SNP detection and revised the manuscript. RZ provided Solexa sequencing data, provided insights on software development and critically revised the manuscript. ZY provided insights on software development and critically revised the manuscript. SS, XL and YZ supervised the project and were involved in revising the manuscript. All authors read and approved the final manuscript.

## Note

Other papers from the meeting have been published as part of *BMC Bioinformatics *Volume 10 Supplement 15, 2009: Eighth International Conference on Bioinformatics (InCoB2009): Bioinformatics, available online at http://www.biomedcentral.com/1471-2105/10?issue=S15.

## References

[B1] Li H Ruan J Durbin R Mapping short DNA sequencing reads and calling variants using mapping quality scores Genome Res 2008 18 1851 1858 10.1101/gr.078212.108 18714091 PMC2577856

[B2] Li RQ Li YR Kristiansen K Wang J SOAP: short oligonucleotide alignment program Bioinformatics 2008 24 5 713 714 10.1093/bioinformatics/btn025 18227114

[B3] Jiang H Wong WH SeqMap: mapping massive amount of oligonucleotides to the genome Bioinformatics 2008 24 20 2395 2396 10.1093/bioinformatics/btn429 18697769 PMC2562015

[B4] Rumble SM Lacroute P Dalca AV Fiume M Sidow A Brudno M SHRiMP: accurate mapping of short color-space reads PLOS Comput Biol 2009 5 5 e1000386 10.1371/journal.pcbi.1000386 19461883 PMC2678294

[B5] Kent WJ BLAT-The BLAST-like alignment tool Genome Res 2002 12 656 664 11932250 10.1101/gr.229202 PMC187518

[B6] Wu TD Watanabe CK GMAP: a genomic mapping and alignment program for mRNA and EST sequences Bioinformatics 2005 21 9 1859 1875 10.1093/bioinformatics/bti310 15728110

[B7] Bona FD Ossowski S Schneeberger K Ratsch G Optimal spliced alignments of short sequence reads Bioinformatics 2008 24 16 i174 i180 10.1093/bioinformatics/btn300 18689821

[B8] MapNext homepage http://evolution.sysu.edu.cn/english/software/mapnext.htm

[B9] Navarro G Raffinot M Flexible Pattern Matching in Strings: Practical On-line Search Algorithms for Texts and Biological Sequences 2001 Cambridge University Press

[B10] Altshuler D Pollara VJ Cowles CR Van Etten WJ Baldwin J Linton L Lander ES An SNP map of the human genome generated by reduced representation shotgun sequencing Nature 2000 407 513 516 10.1038/35035083 11029002

[B11] Brockman W Alvarez P Young S Garber M Giannoukos G Lee WL Russ C Lander ES Nusbaum C Jaffe DB Quality scores and SNP detection in sequencing-by-synthesis systems Genome Res 2008 18 763 770 10.1101/gr.070227.107 18212088 PMC2336812

[B12] Hudson RR Generating samples under a Wright-Fisher neutral model Bioinformatics 2002 18 337 8 10.1093/bioinformatics/18.2.337 11847089

[B13] Rambaut A Grassly NC Seq-gen: An application for the monte carlo simulation of dna sequence evolution along phylogenetic trees Comput Appl Biosci 1997 13 235 238 9183526 10.1093/bioinformatics/13.3.235

